# Antioxidative and osmoprotecting mechanisms in carrot plants tolerant to soil salinity

**DOI:** 10.1038/s41598-022-10835-3

**Published:** 2022-05-04

**Authors:** Iwona Kamińska, Aneta Lukasiewicz, Magdalena Klimek-Chodacka, Olga Długosz-Grochowska, Julia Rutkowska, Kamil Szymonik, Rafal Baranski

**Affiliations:** 1 Department of Botany, Physiology and Plant Protection, Faculty of Biotechnology and Horticulture, University of Agriculture in Krakow, AL. Mickiewicza 21, 31-120 Kraków, Poland; 2 Department of Plant Biology and Biotechnology, Faculty of Biotechnology and Horticulture, University of Agriculture in Krakow, AL. Mickiewicza 21, 31-120 Kraków, Poland

**Keywords:** Plant physiology, Plant stress responses

## Abstract

Soil salinization is a growing problem for agriculture worldwide and carrot is one the most salt-sensitive vegetable species. However, some varieties are capable of withstanding high salt concentrations due to unknown genetic and physiological mechanisms. The aim of this work was to reveal protecting mechanisms against osmotic and ionic stresses that contribute to salt tolerance in carrot. For this purpose, changes in biochemical traits due to soil salinity occurring in the salt-tolerant and salt-sensitive plants were determined. The obtained results showed that the tolerance of the salt-tolerant variety was partially determined constitutively, however, the exposition to saline soil triggered a physiological response that was more evident in the root than in the leaves. The most noticeable changes were the high increase in the content of osmoprotective proline and other low molecular antioxidants such as glutathione and ascorbic acid, and the decrease in the ratio of reduced to oxidized glutathione forms. These changes imply an efficient operation of the ascorbate–glutathione cycle that together with a high activity of antioxidative enzymes such as peroxidases, indicate on the induction of mechanisms associated mainly with protection against excessive reactive oxygen species.

## Introduction

Soil salinity restricts the growth and development of most crop plants and thus contributes significantly to the decrease of yield and quality of agricultural products. More than one billion hectares of lands worldwide is salt-affected^1^ and arable land salinity increases by up to two million hectares per year mainly due to farmland irrigation without adequate soil drainage and using poor quality water^2,3^, and therefore soil salinization affects an estimated 20% of irrigated area^4^. In addition, global climate change and extreme weather conditions, especially rising average temperatures and droughts, contribute to soil salinization^5^. According to a recent report, four billion people (52% of world population) living in 13 countries are affected by soil salinity, and this population may reach five billion assuming actual natural growth by 2050^6^. Therefore, mitigating the effects of arable land salinity poses a challenge for ensuring global food security.

Breeding high yielding crop plants tolerant to abiotic stresses is an effective strategy of sustainable agricultural production in safeguarding food supplies^7^. The recognition of physiological mechanisms and understanding their regulation are critical for the development of stress-tolerant plants using either conventional methods or aided by bio-technological approaches^8,9^. Plants have developed several complex mechanisms ensuring self-protection against elevated salt concentration in soil. Munns and Tester^10^ distinguished two main phases of plant physiological response to salt stress. The fast, or osmotic, phase occurs first due to water deficiency caused by the osmotic effect of salt in the soil. The slow (delayed), or ionic, phase occurs due to the toxic effect of salt ions uptaken by the plant from the soil and accumulated in large amounts. Osmotic and ionic stresses cause also nutritional imbalance and consequently alert the whole metabolism of the plant. Salinity triggers complex signal transduction network of osmotic stress, functioning of ion channels, water and ion uptake and their transport leading to restricted transpiration and disruption of photosynthesis. Ionic stress caused by excess accumulation of Na^+^ and Cl^−^ disturbs the K^+^/Na^+^ homeostasis, hence, affects cell metabolism. The ionic stress signal is perceived by plasma membrane through calcium-dependent protein kinase pathway, known as the SOS (Salt Overly Sensitive) pathway and high-affinity potassium transporter 1^11^, and leads to the secondary stress—oxidative stress, thus, elevated levels of reactive oxygen species (ROS) occur. ROS production induced by salt stress is considered one of the major constraints limiting plant growth and productivity in saline soils^12^. There are several forms of ROS; singlet oxygen (^1^O_2_), hydrogen peroxide (H_2_O_2_), nitrogen oxide (NO) and nitrogen dioxide (NO_2_) are examples of ROS that may release free radicals such as superoxide anion (O_2_^⋅−^), hydroxyl radical (OH^⋅^) and perhydroxyl radical (HO_2_^⋅^)^13,14^. Due to their chemical structures, ROS are highly unstable and reactive and can initiate radical chain reactions that can cause peroxidation of membrane lipids, proteins deactivation, and nucleic acid damage. Changes in the physical properties of cell membranes affect the activity of membrane enzymes and transporting proteins, and lead to loss of selectivity and ultimately to ion leakage or transport disruptions^15–18^.

ROS defence strategy engages enzymatic components and non-enzymatic antioxidants, which serve antioxidative defence against ROS excessive accumulation. Enzymatic machinery is comprised of several enzymes: (1) oxidoreductases: (a) acting on superoxide (superoxide dismutase—SOD), or (b) a peroxide as acceptor (peroxidises: catalase—CAT, ascorbate peroxidase—APX, guaiacol peroxidase—GPOX, glutathione peroxidase—GPx), or c) acting on NADH/NADPH and sulphur group of donors (monodehydroascorbate reductase—MDHAR, dehydroascorbate reductase—DHAR, glutathione reductase—GR) and (2) transferases—glutathione S-transferases (GST). CAT and SOD catalyse dismutation reaction of H_2_O_2_ and O_2_^⋅−^, respectively. APX, MDHAR, DHAR and GR are component of ascorbate–glutathione cycle, which is an effective metabolic pathway of H_2_O_2_ detoxification^19,20^. GPx, some GST and peroxidases neutralize H_2_O_2_ and organic hydroperoxides through ascorbate-independent thiol-mediated pathways using nucleophiles such as reduced glutathione (GSH), thioredoxin (TRX) or glutaredoxins (GRX)^21,22^. Increased activity and transcript levels of antioxidant enzymes were observed as the response to salinity and can determine greater resistance to stress^23^.

The activity of antioxidant enzymes depends on the availability of low-molecular antioxidants, mostly glutathione and ascorbic acid^24^. Other nonenzymatic antioxidants present in plant cells are proline, flavonoids, carotenoids, tocopherols and glycine betaine^13,25^. Through their multifunctional activity in a cell, low-molecular antioxidants can serve as protection guards for stress-induced disruptions of the metabolism. Glutathione is responsible for cell membranes and proteins protection; it modifies the activity of enzymes, plant hormones or gene expression. It is also a signal molecule that activates redox signal transduction. The physiological role of glutathione is also associated with toxic metals chelation, which keeps them in vacuole system^26^. The antioxidative role of ascorbic acid (ASC), apart of its involvement in ascorbate–glutathione pathway, is related with direct detoxification of ROS and the regeneration of α-tocopherol (vitamin E)^27,28^. Proline (Pro), the aromatic amino acid, as a small osmotically active molecule, protects cells from osmotic outflow of water and thus, prevents the cell dehydration^29–31^. It also participates in stabilizing cellular structures (mainly membranes and proteins) or in scavenging free radicals. Pro can be involved in gene expression induction associated with the plant response to salt stress^32,33^. Phenolic compounds are a large group of secondary metabolites. They possess antioxidant activity, play role of signal molecules (salicylic acid), inducing the expression of genes associated with secondary metabolism (e.g. cell wall strengthening by lignins). Moreover, phenolics, due to their possibility of binding with sugars enable their transport, and thus regulate the osmotic pressure of cells^11,34,35^.

Carrot (*Daucus carota*) is a glycophyte and grows in soils with a low content of sodium salts^36^ but there exists a variation in susceptibility to salinity among carrot accessions, and the carrots growing in a highly saline soil have been reported^37^. Salt-stressed carrot plants show inhibited growth, morphological abnormalities, and accumulation of membrane lipid degradation products such as malondialdehyde (MDA). At biochemical level, they exhibit decreased soluble proteins content and lower activity of SOD, CAT and peroxidase (POD). Plants with enhanced tolerance to salinity may respond with morphological changes having denser distribution of trichomes on leaves and petioles, induced accumulation of anthocyanins in petioles, and higher accumulation of glycine betaine, and ascorbic acid in roots^38–40^. Song and Ahn^41^ correlated carrot tolerance towards salinity with salt stress-generated expression of small heat shock protein. Salt stress response of carrot is affected by abscisic acid (ABA) hence regulation through carotenoid biosynthesis pathway was postulated^42^. Genetic engineering led also to the development of carrot with enhanced osmoprotective mechanisms and withstanding salinity of 400 mM NaCl in soil^43^.

Recently, a comprehensive analysis of element uptake, transport and distribution in the root and leaves of plants exposed to saline soil has been reported^44^, in which, we compared two carrot varieties: the DH1 line of Nantes type (a common western carrot type grown in Europe and North America) and the DLBA landrace of eastern carrot type originating from region with saline soil. Salt stress limited plant growth of both varieties but for the DH1 plants, the leaves showed chlorosis and wilting, and they withered faster than the DLBA leaves, and the root biomass reduction due to soil salinity was about 40% more severe than for DLBA. Hence, as expected, the DH1 plants were highly sensitive to salinity while the DLBA variety performing better showed some degree of tolerance. This tolerance of the DLBA plants was manifested also by their higher ability to uptake and increased transport of Na^+^ and Cl^−^ to the leaves, and higher accumulation of these elements in the roots and leaves. Hence, detoxification of high Na and Cl amounts was more effective in DLBA. This ability corresponded to a higher selective uptake of other elements, including K, and to an effective K transport up to the leaves. We also found in the DLBA leaves the unique expression of DcNHX4 cation:proton exchange transporter presumably participating in monovalent cation transport through tonoplast thus in maintaining K^+^ homeostasis in the cytosol of salt-stressed plants^44^.

The above-described capability for ion compartmentalization and the expression of one antiporter may only partially explain salt tolerance of the DLBA plants. Undoubtedly, other physiological processes supporting water uptake and mitigating toxic effects of excessive Na^+^ and Cl^−^ ions must function in the tolerant DLBA plants growing in the saline soil. Thus, the aim of the present work was to reveal potential mechanisms protecting against osmotic and ionic stresses that contribute to salt tolerance in carrot. For this purpose, we evaluated changes in biochemical traits occurring in the salt-tolerant DLBA and salt-sensitive DH1 plants due to soil salinity. We describe different responses of these varieties and indicate mechanisms related mainly to protection against excessive ROS, prevailing in either the roots or leaves.

## Results

**Figure 1:**
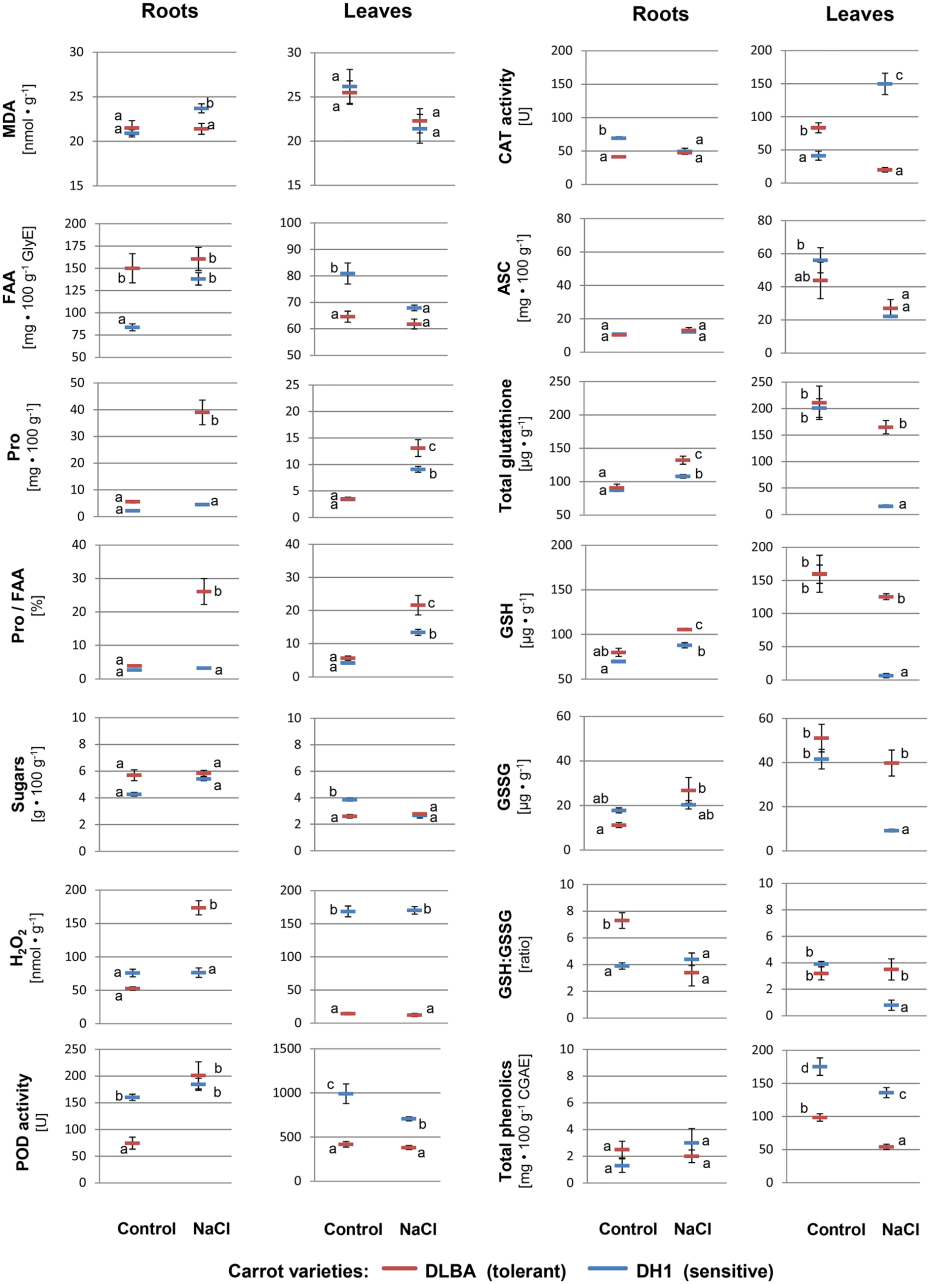
Interaction plots. Changes in the mean contents of biochemical components and in enzyme activities in the salt-tolerant DLBA and salt-sensitive DH1 carrot varieties growing for 102 days in the saline soil (EC = 3.0 dS m^−1^) in comparison to the control (EC = 0.2 dS m^−1^). Means marked by the same letter within a plot do not differ at *P* = 0.05 significance level according to the Newman–Keuls multiple comparison test. Whisker—standard error of the mean. For abbreviations see Table 1.

Roots and leaves of salt-tolerant DLBA and salt-sensitive DH1 carrot plants, growing in either the control or saline soil, were assessed with regard to the content of biochemical compounds and enzyme activity. In total 24 parameters were determined that allowed comparison of the two varieties and how they reacted to salt stress. A two-way analysis of variance revealed that varieties differed in most of these traits and the salt stress changed the level of compounds or enzyme activity in both, roots and leaves (Table 1). Moreover, DLBA and DH1 plants reacted in a different way to the applied salt stress as indicated by significant interaction effects (Table 1), which were visualized in interaction plots (Fig. 1).

**Table 1:** Significance levels obtained in two-way analyses of variance applied to 24 biochemical traits evaluated independently in the roots and leaves of salt-tolerant DLBA and salt-sensitive DH1 carrot plants growing for 102 days in the control (EC = 0.2 dS/m) and saline soil (EC = 3.0 dS/m).

Trait (abbreviation)	Roots			Leaves		
	Variety (V)	Salt stress (S)	V × S	Variety (V)	Salt stress (S)	V × S
Relative water content (RWC)	–	–	–	0.846	** < 0.001**	0.243
Malondialdehyde content (MDA)	0.189	**0.030**	**0.022**	0.947	**0.017**	0.632
Proline content (Pro)	** < 0.001**	** < 0.001**	** < 0.001**	**0.020**	** < 0.001**	**0.031**
Free amino acids content (FAA)	** < 0.001**	**0.006**	0.058	** < 0.001**	**0.003**	**0.047**
Pro/FAA ratio (Pro/FAA)	** < 0.001**	** < 0.001**	** < 0.001**	**0.004**	** < 0.001**	**0.038**
Sugars content (sugars)	0.114	0.266	0.385	** < 0.001**	** < 0.001**	** < 0.001**
H_2_O_2_ content (H_2_O_2_)	** < 0.001**	** < 0.001**	** < 0.001**	** < 0.001**	0.971	0.725
Peroxidase activity (POD)	**0.036**	** < 0.001**	**0.003**	** < 0.001**	**0.016**	0.060
Catalase activity (CAT)	** < 0.001**	**0.034**	** < 0.001**	** < 0.001**	**0.010**	** < 0.001**
Ascorbic acid contnet (ASC)	0.696	**0.019**	0.393	0.621	**0.004**	0.258
Total glutathione content (Glutath._total)	**0.011**	** < 0.001**	**0.043**	**0.005**	** < 0.001**	**0.010**
Reduced glutathione content (GSH)	**0.002**	** < 0.001**	0.271	**0.009**	**0.001**	**0.010**
Oxidized glutathione content (GSSG)	0.988	**0.009**	**0.041**	**0.003**	**0.002**	0.071
GSH/GSSG ratio (GSH/GSSG)	0.059	**0.011**	**0.003**	0.061	**0.016**	**0.006**
Total phenolics contnet (PhC_total)	0.893	0.428	0.119	** < 0.001**	** < 0.001**	0.760
Phenylpropopanoids content (Phenylprop.)	0.242	0.695	0.548	** < 0.001**	** < 0.001**	0.841
Flavonols content (Flavonols)	0.219	0.255	0.843	**0.001**	**0.001**	0.787
Anthocyanins content (Anth.)	0.686	0.542	0.777	0.419	0.434	0.728
Phenylpropanoids/PhC-total (%_Phenylprop.)	0.786	0.068	0.327	** < 0.001**	**0.038**	0.165
Flavonols/PhC-total (%_Flavonols)	0.451	0.271	0.245	** < 0.001**	0.066	0.684
Anthocyanins/PhC-total (%_Anthoc.)	0.366	0.500	0.871	**0.006**	0.204	0.185
*p*-hydroxybenzoic acid (*p*-HBA)	** < 0.001**	0.143	0.110	0.492	0.103	**0.014**
Chlorogenic acid content (CGA)	**0.030**	**0.014**	**0.003**	** < 0.001**	0.080	0.078
Ferulic acid contnet (FeA)	–	–	–	** < 0.001**	0.181	0.181

Significant levels below 0.05 are in bold (n = 5).

### Stress markers

Two stress markers were assessed, a relative water content (RWC) and MDA content. The DLBA and DH1 plants growing in the control soil had similar RWC (90% and 88%, respectively) in the leaves. In saline soil, they had lower hydration in comparison to the control by 14% and 10%, respectively. The content of MDA in the roots of control plants was similar in both varieties (21 nmol g^−1^ FW) but in the salt-stressed plants the MDA content increased by 13% in DH1 but did not change in DLBA. In leaves of both varieties, the MDA content decreased by 13–18%.

### Osmoprotectants

The content of free amino acids (FAA) in roots and leaves differentiated varieties and was affected by soil salinity. Salt stress caused 65% increase of the FAA content in the roots and 16% decrease in the leaves of DH1 plants but no significant changes were found for DLBA. In contrast, the Pro content increased in roots and leaves of both varieties exposed to salt stress however with different rate. The most pronounced, sevenfold increase, was observed in the DLBA roots. In the DH1 roots, a twofold increase was not confirmed to be significant. In the leaves of control plants, the Pro content was at a similar level as in the roots, and it increased 3.8 and 2.7 times under salt stress in DLBA and DH1, respectively. The Pro content expressed as the percentage of total FAA content was at a similar level (2.6–5.2%) in both varieties when plants were growing in the control conditions. Salinity caused that the Pro contribution increased 6.7-fold in the DLBA roots reaching 26.1% of FAA while it did not change in DH1. Hence, the Pro contribution to FAA was eight times higher in the roots of salt-stressed DLBA plants than in DH1. In the leaves, in stress conditions, the Pro contribution to FAA increased 3.9-fold and 3.2-fold for DLBA and DH1, respectively, and was 1.6 times higher in the DLBA than in DH1.

The contents of soluble sugars (reducing and non-reducing) were similar in the roots of both varieties (4.3–5.7 g 100 g^−1^ FW) and they were not affected by salt stress. The leaves of DH1 control plants contained more sugars (3.9 g 100 g^−1^ FW) than DLBA (2.6 g 100 g^−1^ FW). Salt stress reduced the sugars content in DH1 by 31%, so their amount was the same as in the DLBA leaves. No change was observed for DLBA. The sugar content in the whole plant was similar in both varieties and not affected by stress treatment but a higher content in the roots than in leaves of DH1 indicated for sugars relocation.

### H_2_O_2_ and antioxidative enzymes

The H_2_O_2_ amount in the roots of control plants was similar in both varieties but a high increase (3.3-fold) was observed in DLBA growing in the saline soil, unlike in DH1. In the leaves, salt stress did not affect H_2_O_2_ content in any variety but the level of H_2_O_2_ in DLBA was about 12-fold lower than in DH1.

Carrot varieties exhibited different activities of antioxidative enzymes. The POD activity was lower in the DLBA roots and leaves than in DH1. It was twofold lower in the roots of control DLBA plants than in DH1, and it increased 2.7 times in saline conditions reaching the level of POD activity found in DH1. The POD activity remained unaffected in the DH1 roots. In the leaves of control plants, the POD activity was six times higher than in the roots. In saline conditions, the POD activity remained unaltered in the DLBA leaves while it decreased by 30% in DH1.

Catalase activity also highly depended on variety, the applied stress and their interaction, for both, roots and leaves. In the DLBA roots, CAT activity was lower than in DH1 and did not change under salt stress while in DH1 it decreased by 28% to the level found in DLBA. In the leaves, the variety × salt interaction was different. Salt stress reduced CAT activity fourfold in DLBA and increased 3.6 times in DH1. Hence, CAT activity in the leaves of salt-stressed DLBA plants was almost eight times lower than in DH1.

### Low molecular weight antioxidants

The contents of low molecular weight antioxidants such as glutathione, ASC and phenolics were also assessed. The control plants of both varieties did not differ in the total glutathione content, although leaves contained over doubled amount of glutathione than roots. A ratio of a reduced to oxidized glutathione form (GSH/GSSG) was two times higher in DLBA than in DH1 roots, mainly due to 40% lower GSSG content and 15% higher GSH content. The application of salt stress increased the glutathione content in a different way depending on the variety. In the DLBA roots, the GSH, GSSG and total glutathione contents increased by 32%, 139% and 46%, respectively, while in DH1 their increase was less evident, 26%, 14% and 24%, respectively. In consequence, in DLBA, the glutathione contents were 1.2–1.3 times higher than in DH1 and GSH/GSSG ratio of 7.3 decreased by 53% to 3.4 while in DH1 the ratio remained unchanged at 3.9–4.4 level (a non-significant increase). In the leaves, both glutathione forms and its total pool decreased but in DLBA the change was small (28% decrease, independent on the form) in comparison to DH1 where glutathione was present in very small amounts (96%, 78% and 92% reduction for GSH, GSSG and total glutathione, respectively, in comparison to the amounts determined in the leaves of control DH1 plants). Hence, in DLBA, the glutathione contents were 4–19 times higher (depending on the form) than in DH1 and the GSH/GSSG ratio remained unchanged.

The content of ASC in the roots of control plants was low (at the level of 10 mg 100 g^−1^ FW), and increased under salt stress by 26% and 12% in DLBA and DH1, respectively, although such changes were not confirmed to be significant. In the leaves of control plants, the ASC content was 44–56 mg 100 g^−1^ FW and it decreased due to the salt stress. The reduction was more pronounced in DH1 (61%) than in DLBA (38%).

The total amounts of phenolic compounds were very low in the roots *i.e*., below 3 mg 100 g^−1^ FW, independent on the variety and treatment. In the leaves of control DLBA and DH1 plants, 98.5 and 175.3 mg 100 g^−1^ of phenolics were present and their amounts decreased in stress conditions by 44.4 mg 100 g^−1^ and 39.3 mg 100 g^−1^, respectively, hence, the change was similar in both varieties. Phenylpropanoids dominated in the DH1 leaves (49% of total phenolics), followed by flavonols (44%) and anthocyanins (7%), and similar proportions were maintained in the salt-stressed plants. In the DLBA, these compound groups constituted 39–42%, 50–51% and 8–10%, respectively. Hence, flavonoids (flavonols and anthocyanins) predominated in DLBA (about 60%) while in DH1 phenylpropanoids and flavonoids occurred in similar amounts.

Chlorogenic acid (CGA) was one of the dominating phenolic compound, and its content decreased in the DH1 leaves under stress conditions by 32% to the level of 105 mg 100 g^−1^. The content of *p*-hydroxybenzoic acid (*p*-HBA) also increased by 58% but ferulic acid (FeA) content decreased by 45%. Both compounds occurred in much lower amounts than CGA, at the level of 1 mg 100 g^−1^ and 10 mg 100 g^−1^, respectively. In the DLBA leaves, CGA was present in very low amounts (below 5 mg 100 g^−1^) in comparison to DH1, and FeA was not detected. No changes in CGA and *p*-HBA contents were found under salt stress.

### Multidimensional approach

**Figure 2:**
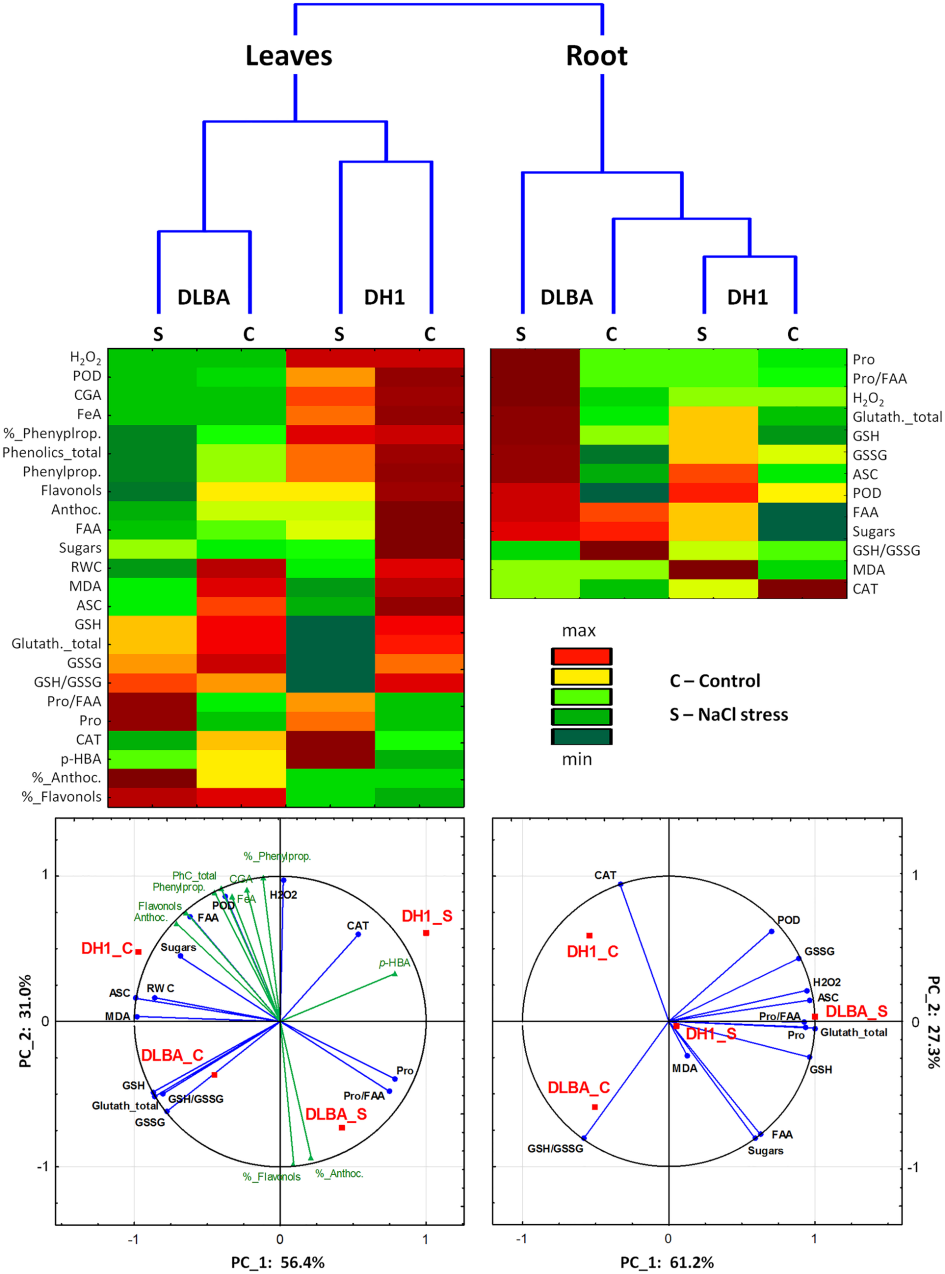
Cluster analysis. Dendrograms obtained from hierarchical cluster analysis using Euclidean distances and Ward amalgamation, two-way joining maps, and biplots obtained from principal component analysis of biochemical parameters for salt-tolerant DLBA and salt-sensitive DH1 carrot varieties growing for 102 days in the control (EC = 0.2 dS m^−1^) and saline soil (EC = 3.0 dS m^−1^). For abbreviations see Table 1.

Multidimensional data analyses were performed to summarize differences between varieties in their response to salt stress as well as to identify the contribution of biochemical parameters to this response. Most traits differed greatly between the roots and leaves, and cluster analysis confirmed that plant part was the most discriminating factor (Fig. 2). In general, the content of FAA, Pro and sugars were higher in the roots while most other traits had higher values for leaves. Carrot variety was the second order discriminating factor and the plant parts of the control and salt-stressed plants of the same variety grouped together, which indicated that changes due to the applied stress were less evident than differences between DLBA and DH1 or that their responses to salt stress were different.

Principal component analysis applied to root data visualized contribution of 13 traits to overall variation when two varieties in the control and stress conditions were compared (Fig. 2). Phenolic compounds were excluded from the analysis as they appeared in the roots in low amounts only and had no confirmed effects. Almost all traits were very well represented in a two-dimensional scatter plot. The first two principal components, PC1 (61.2%) and PC2 (27.3%), explained 88.5% of total variation and the remaining 11.5% was explained by the PC3. The proline content and percentage contribution of Pro to FAA, H_2_O_2_, ASC, GSH, GSSG and total glutathione contents were the most correlated (r = 0.90–0.99) variables with PC1. The addition of PC2 enabled visualization of changes mainly in CAT activity, GSH/GSSG ratio, sugars and FAA contents; absolute correlation values for these traits with PC2 were above 0.8 and total loadings to PC1 and PC2 raised above 0.97. Only POD activity was partially correlated with both, PC1 (r = 0.70) and PC2 (r = 0.62). PC3 almost exclusively represented MDA content (r = 0.96). Visualization of relationships between varieties and their biochemical parameters in a biplot was highly congruent with a heat map generated using the two-way clustering analysis (Fig. 2). PC2 differentiated mainly between DLBA and DH1 growing in the control conditions. The control DLBA roots, in contrast to DH1, were characterized by higher GSH to GSSG ratio, and sugars and FAA contents while CAT and POD activities, and GSSG contents were lower. PC1 explained mainly the response of both varieties to salt stress. Salinity induced changes that were much more pronounced in DLBA than in DH1. The roots of salt-stressed DLBA plants had a higher Pro contribution to FAA, increased contents of Pro, H_2_O_2_, total glutathione, GSH, GSSG, ASC, and POD activity while GSH/GSSG ratio was decreased. Such changes were much less evident or not observed in DH1. In contrast, the increase of MDA, FAA and sugars contents, and the decrease in CAT activity were the most noticeable in the response of salt-stressed DH1 roots.

Principal component analysis applied to the leaf data visualized contribution of the same 13 traits as for the roots and additionally, RWC and 11 traits referring to phenolic compounds. The first two principal components PC1 (56.4%) and PC2 (31.0%) explained 87.4% of total variation. MDA and ASC contents were almost exclusively represented by PC1 (both r = -0.99); other highly correlated parameters with PC1 were: RWC, those related to the glutathione pool, Pro content and Pro contribution to FAA (r = 0.56–0.76). The addition of PC2 enabled visualization of changes in H_2_O_2_ and FAA contents, and POD activity, with total loadings to PC1 + PC2 of 0.89–0.95. Phenolic compounds contents were represented also mainly by PC2 (loadings to PC2 only were 0.79–0.98) with the exception of *p*-HBA being highly correlated mainly with PC1 (r = 0.79), and flavonols and anthocyanins contents equally well represented by both PCs (loadings to PC1 + PC2 = 0.95–0.98). PC3 explaining only 12.6% of total variation was mainly correlated with CAT activity, sugars and *p*-HBA contents (r = 0.52–0.60, absolute values).

PC2 discriminated between the DLBA and DH1 varieties. The leaves of control DLBA plants had either similar or lower values of measured parameters than the leaves of control DH1 plants, with the exception for the GSSG content, CAT activity, *p*-HBA content and percentage of flavonols and anthocyanins in total phenolics. PC1 discriminated plants growing in the control and stressed conditions. The DLBA plants responding to salt stress had a higher Pro content and its contribution to total FAA. Other major changes observed in the DH1 leaves such as lower ASC and glutathione pool contents were less evident in DLBA. The CAT activity and *p*-HBA content strongly increased in DH1 while they decreased in DLBA.

## Discussion

As have been shown earlier^44^, both varieties, DLBA and DH1, exhibit adverse symptoms occurring after the exposition to salt stress by developing smaller organs, in particular roots, and lower biomass. Such reaction was less evident for the DLBA plants justifying this variety to be considered tolerant to salinity. The results of present work reveal other adverse effects at biochemical and physiological levels occurring in the salt-stressed carrot plants. Water uptake by plants growing in the saline soil is limited due to osmotic force that leads to plant, and in particular to leaf, dehydration. In both DLBA and DH1 varieties salt stress caused water management disruptions. The leaf RWC decrease was however similar for the plants of tolerant and sensitive varieties. A strong RWC decrease in leaves is usually observed in sensitive plants^45–48^ while the accumulation of osmoprotectants is considered as an adaptive mechanism enhancing the succulence and securing maintenance of water balance. Increased Pro content acts as the osmotic adjustment mechanism towards prevention of cell dehydration; this small osmotically active molecule inhibits the loss of water from plant cells due to its osmotic outflow^29–31^. Such mechanism however may not be sufficient against long-term salinity-induced osmotic stress^49–51^. Both carrot varieties accumulated osmoprotectants (Pro) and DLBA plants had much higher Pro levels than DH1. The decrease of RWC in the DLBA and DH1 leaves was however similar suggesting that both varieties did not differ with regard to the efficiency of mechanisms preventing dehydration. This does not exclude the option that water uptake and transpiration are more intense in the DLBA than in DH1 plants, which would be required for higher biomass production by DLBA.

Another common biochemical marker of injuries caused by salinity is the occurrence of MDA, the final product of lipid peroxidation due to cell membrane damage^52,53^. Reactive oxygen species, which occur in oxidative stress, attack membrane phospholipids and change their physicochemical properties, which affects membrane permeability, transmembrane transport, and metabolic processes. Salinity stress, causing oxidative stress within the cells, may thus also lead to peroxidation of membrane lipids^52^, and higher amounts of MDA are often found in plants sensitive to salinity^54,55^. The MDA contents were elevated in the DH1 roots due to salt stress while in the DLBA plants the MDA levels did not change. This result indicates that lipids in the DH1 root cells are prone to ROS attack while mechanisms neutralizing ROS are either more active or induced in DLBA.

The comparison of biochemical traits shows that the examined carrots exhibited variety- and organ-specific response to soil salinity. The differences between the tolerant DLBA and sensitive DH1 varieties can be recognized as those that are constitutive and those, which are induced by stress. Constitutive differences could be identified when the DLBA and DH1 plants growing in the control, non-saline soil were compared. The DLBA plants had lower enzyme activity, higher FAA content and higher GSH/GSSG ratio in the roots than the DH1 plants. In the leaves, DLBA had much lower H_2_O_2_ contents, much lower POD activity but higher CAT activity, and lower amounts of phenolics but higher share of flavonoids. Hence, different physiological activities were observed in the leaves and roots of non-stressed plants, and, in general, DLBA was characterized by lower trait values.

The application of salt stress revealed meaningful differences in the response of both varieties. In the root, *i.e.*, in the organ directly exposed to stress, salinity induced changes in the Pro and FAA levels. Despite Pro is an osmoprotectant, it participates in stabilizing cellular structures (mainly membranes and proteins) and in scavenging of free radicals. Some studies also report the role of Pro in neutralizing low pH of the cytoplasm and maintaining NADP+ to NADPH homeostasis^56^. Up-regulation of antioxidant enzyme gene expression by Pro was also postulated^33,57^. The increase in Pro contents was one of the most noticeable reaction of carrot plants exposed to saline soil and that could be associated with Pro role in osmotic adjustment. The tolerant DLBA plants had almost nine times higher Pro contents in the roots than DH1 and Pro consisted 1/4 of all FAA in the DLBA roots. This indicates high activity of Pro biosynthesis as an immediate response and prevailing in the salt-tolerant DLBA plants. Similar trend, although less manifested, was found in the leaves of stressed plants; hence, the tolerance mechanism involving Pro accumulation was activated in the whole plant. Notably, in the non-saline soil, the DLBA roots seemed to have also a higher Pro content and higher Pro contribution to FAA than the DH1 roots. This suggests that DLBA possesses a constitutively enhanced Pro biosynthesis, which could prerequisite DLBA to withstand elevated salinity in the soil.

It seems also that osmotic protection in the DH1 roots might be supported by sugars accumulation, although the results do not allow for unambiguous conclusion. A tendency for enhanced relocation of sugars from the leaves to root was observed in DH1 exposed to saline soil while the total sugar content in the plant remained stable. Such changes were not found in DLBA showing a tendency for higher sugar formation that is congruent with better DLBA adoption to salt-stress conditions thus higher biomass production.

Salt stress generates oxidative stress through depletion of antioxidants or accumulation of ROS. Cells attempt to counteract the oxidant effects and restore the redox balance, which in turn gives rise to antioxidant constituents responsible for eradicating excessive ROS^18^. Tolerance to salinity is often associated with H_2_O_2_ level within the tissues^58–61^. This key signalling molecule controls physiological responses to biotic and abiotic stresses such as drought, salinity, cold and high temperature^62,63^ but at high concentrations, it causes lipid peroxidation, protein and DNA damages^64^. Exogenous H_2_O_2_ treatment can lead to a significant accumulation of Pro due to a rapid increase of the glutamate pathway activity^65^. In turn, Pro may be involved in scavenging free radicals and neutralizing H_2_O_2_ excess^66,67^. This mechanism is apparently also active in the salt-tolerant carrot variety as both H_2_O_2_ and Pro contents increased significantly in the DLBA roots. While H_2_O_2_ amounts did not change in the DH1 roots exposed to the saline soil, it remains inconclusive whether H_2_O_2_ level is preserved effectively by Pro and other defence mechanisms, or signal pathways with H_2_O_2_ contribution are not induced. Altogether, the results indicate that proline biosynthesis genes are potential genetic determinants involved in carrot adaptation to saline soil or response to salt stress. Proline is synthesized from glutamate in reactions conferred by Δ^1^-pyroline 5-carboxylate (P5C) synthase followed by P5C reductase^68^. Alternatively, the proline precursor, P5C, can be synthesized via the arginine and ornithine pathway that extends a list of potentially up-regulated genes in carrot tolerant plants. Moreover, proline is catabolised by proline dehydrogenase back to P5C and then by P5C dehydrogenase to glutamate^69^. It is also metabolised to 4-hydroxyproline by proline hydroxylase^70^, so down-regulation of these genes can also be an alternative mechanism affecting proline accumulation.

Enzymes catalysing the reactions of radical neutralization and decomposition of excessive ROS, mainly O_2_^·−^ and H_2_O_2_, such as superoxide dismutase (SOD), catalase (CAT), peroxidase (POD) with cooperation with reductases are highly effective enzymatic defence system from damages caused by oxidative stress. Superoxide anion radical molecules are dismutated by SOD to less reactive H_2_O_2_. POD and CAT are responsible for the removal of H_2_O_2_ by its further breakdown into O_2_ and H_2_O^71^. High increase of POD activity was observed in the roots when the plants of tolerant DLBA were exposed to the saline soil. Considering that H_2_O_2_ level in these roots also raised in stressed conditions, and the levels of both, H_2_O_2_ and POD, increased by about three times, one can expect that POD was directly involved in defence response to oxidative stress. The roots of salt-sensitive DH1 were not protected by this mechanism. In the leaves, no changes in H_2_O_2_ content and POD activity were found, except POD activity decrease in DH1. Notably, CAT activity was not stimulated in the roots by salt stress in any variety; in the leaves, CAT activity highly decreased in DLBA and highly increased in DH1 while, in both varieties, the H_2_O_2_ levels remained unaffected by the application of salt stress. Hence, there was no correlation between H_2_O_2_ levels and CAT activity in any variety, either in the roots or leaves. It can be postulated that the mechanism involving the generation of H_2_O_2_ signal molecules and their further neutralization by Pro and decomposition by POD can influence carrot tolerance to salinity. Furthermore, H_2_O_2_ are signal molecules in mechanisms preventing damage of DNA, RNA, lipids, proteins, carbohydrates and pigments. Thus, H_2_O_2_ and PODs are involved in a wide range of physiological processes, including cell wall metabolism such as lignification and suberisation^25^. The DLBA plants have much higher ability to accumulate excess of Na^+^ and Cl^−^ than the DH1 plants^44^. These ions can be relocated to vacuoles or, at least partially, immobilized in the cell wall^72^. Strengthening cell wall physical barrier by lignification and suberisation enhances cell wall capacity of Na^+^ and Cl^−^ adsorption^73^. Thus, H_2_O_2_ generation and its further conversion might be an auxiliary mechanism mitigating the toxicity of Na^+^ and Cl^−^ ions operating in the plants of salt-tolerant DLBA variety. Further cell anatomical and ultrastructural observations could validate such hypothesis.

Despite Pro, other low-molecular antioxidants, mostly glutathione and ascorbic acid and phenolic compounds, are involved in antioxidative defence against ROS damages^13^. Glutathione performs a number of functions in plant cells; it is responsible for cell membranes and proteins protection and thus is a part of the detoxifying system of ROS, including neutralization of H_2_O_2_, generated due to salt stress^74^. Glutathione takes part in the maintenance of cell redox potential, which is possible due to a regenerative system converting oxidized glutathione (GSSG) to its reduced form (GSH). This is done with the participation of glutathione reductase, whose activity determines the GSH/GSSG ratio. This ratio determines the structural changes of proteins through interactions with thiol groups (-SH) and disulfide bridges (–S–S–), which in turn regulate enzymes activity^22^. In addition, the redox potential resulting from the GSH/GSSG ratio (but also ASC/DHA or NADPH/NADP) can affect gene expression. Such expression control is observed to the cytosolic SOD or glutathione reductase pool^75^. Glutathione is also a signal molecule that activates redox signal transduction and through that can lead to programmed cell death^76^. Moreover, it is associated with chelation of toxic metals facilitating their compartmentalization^26^. The contents of glutathione and ascorbate did not differentiate the DLBA and DH1 plants growing in the control soil. However, the GSH/GSSG ratio was almost doubled in the DLBA roots in comparison to the DH1 roots that indicates more efficient regenerative system of GSH and higher reduction state, which maintains protein structure and function in DLBA. The total glutathione pool increased in stress conditions but such change was more pronounced in the DLBA roots than in DH1. Moreover, despite effective regenerative system of GSH, the GSH/GSSG ratio decreased in the roots of salt-stressed DLBA plants while in the DH1 roots the ratio remained unchanged. This shift can contribute to the signalling pathways and activation of the defence mechanism involving glutathione. Furthermore, a more pronounced increase in GSSG content found in the DLBA roots indicate that lager amounts of GSH were oxidized as the result of cell protection against oxidative stress. Both, the increased content of glutathione pool and higher GSH involvement support hypothesis that this antioxidant plays an important role in carrot root protection against soil salinity and that its contribution is stronger in the tolerant variety. The aforementioned results indicates that the glutathione reductase gene and others genes participating in GSH↔ GSSG conversion, coding such enzymes as glutathione *S*-transferases, peroxidases and NADP-dependent dehydrogenases, are differentially regulated in the salt-tolerant and salt-sensitive carrots. Taking into account that glutamate is a common substrate for pathways leading to either GSH or proline biosynthesis^77^, the role of genes conferring glutamate availability seems essential. Up-regulation of glutamate synthase and glutaminase, and down-regulation of glutamate dehydrogenase and glutamine synthase may affect the balance between glutamate and glutamine, or between glutamate and its another precursor, 2-oxoglutarate.

GSH is also an electron donor for enzymes involved in maintaining a high ratio of reduced to oxidized ascorbate (ASC/DHA). In turn, ASC is used to detoxify H_2_O_2_. The relationships between these molecules are described in the reaction cycle known as the ascorbate–glutathione cycle (or Asada-Halliwell-Foyer pathway)^19,78^. This cycle maintains the cellular pool of both low-molecular antioxidants in their reduced state and it was found crucial for effective ROS scavenging in a halophyte *Zygophyllum xanthoxylum*^79^. In the roots of tolerant DLBA carrot variety a similar increase of ASC (26%) and GSH (32%) in stress conditions was observed. This indicates no significant disturbances occurred in the ASC-GSH cycle. In the salt-stressed roots of sensitive DH1 line, the increase of ASC content was a twofold lower than in DLBA. Such discrepancy may suggest instability of the ASC-GSH cycle in the salt-sensitive plants. The pool of low-molecular antioxidants, GSH, ASC, and additionally Pro, in the roots increased more in the tolerant than in sensitive variety when the plants were exposed to salt stress. This suggests that the tolerant variety, apart of ASC-GSH cycle, can also activates other glutathione-dependent mechanism of ROS detoxification in the roots but further study is needed to investigate the exact pathways. The difference between carrot varieties in glutathione pool and ASC contents was much more evident in the leaves. Although glutathione content decreased in both varieties, the decrease in the salt-tolerant DLBA was at about 20% level in comparison to the non-stressed plants while in salt-sensitive DH1 glutathione was present in low amounts only which certainly were insufficient to ensure effective protection. In consequence, the decrease in ASC content was also more evident in the sensitive variety.

Phenolic compounds possess antioxidant activity, thus they may protect cell structures against damage from excessive ROS generated under oxidative stress. They also play a role of signal molecules (salicylic acid), inducing the expression of genes associated with secondary metabolism (e.g. cell wall strengthening by lignins). Moreover, due to the affinity to sugars, phenolics enable their transport facilitating the regulation of cell osmotic pressure^11,34,35,80^. The obtained results suggest that the metabolism of phenolic compounds in the leaves of carrot plants subjected to salinity stress is inhibited, regardless of whether the plants exhibit tolerance or not. In the roots, phenolics were present in very low amounts, which did not change in stressed plants. Therefore, the role of phenolics in carrot tolerance to salinity is not justified unless their accessory contribution. In comparison to the sensitive DH1 line, the tolerant DLBA variety was characterized by a slightly lower proportion of phenylpropanoids thus greater proportion of flavonoids in the leaves of plants growing in saline soil. Flavonoids, which include anthocyanins, flavonols and flavones, have more OH groups that makes them more antioxidant active molecules. However, the total level of flavonoids in DLBA remained lower than in DH1. These observations do not exclude a potential role of individual phenolic compounds, such as salicylic acid or its derivative *p*-HBA, signal molecules active also in salt-stressed plants^81^. Apparently, a twofold increase of *p*-HBA was found in the leaves of DH1 plants growing in the saline soil.

## Conclusions

**Figure 3:**
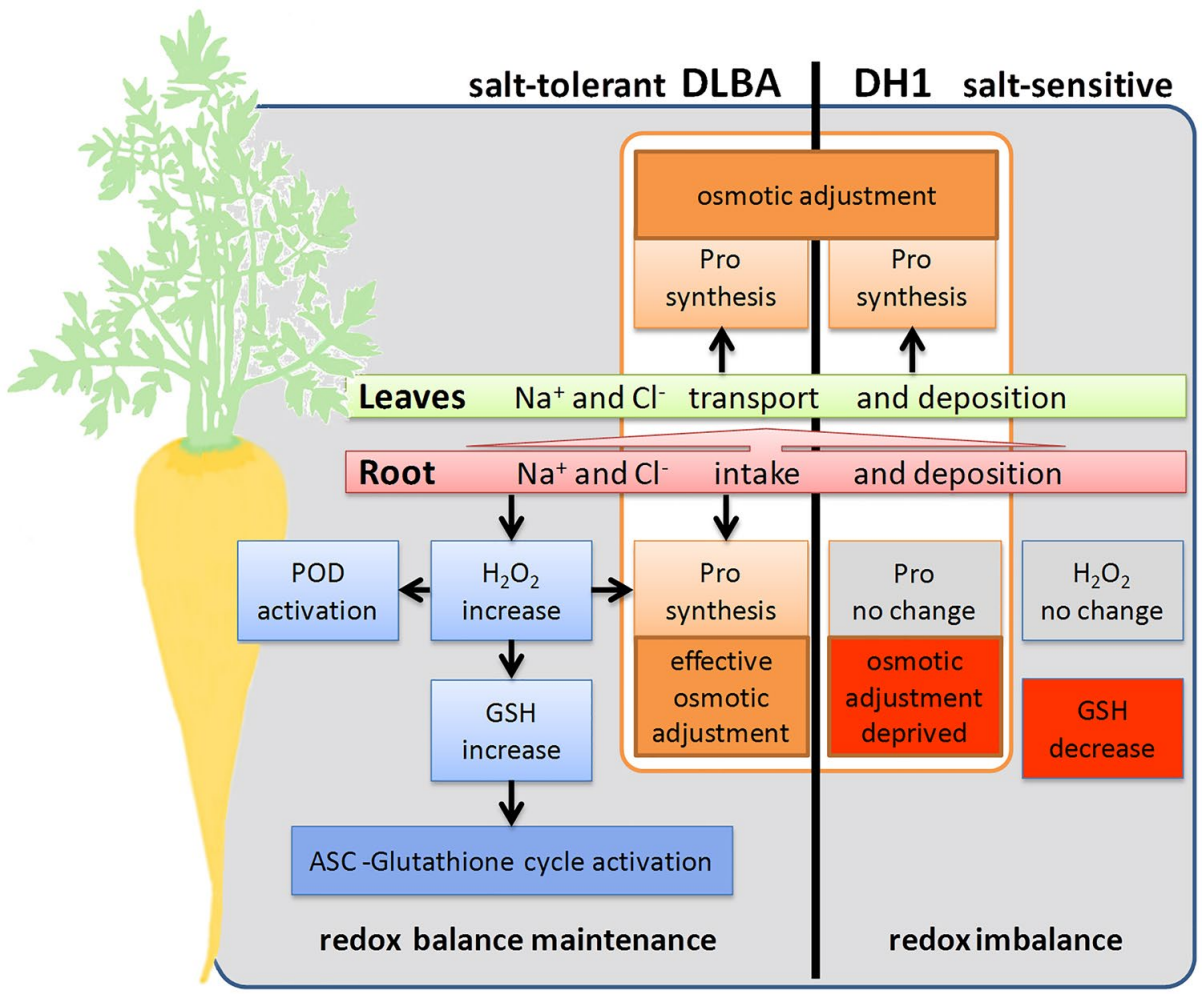
Activation of antioxidant system and osmotic adjustment mechanisms in salt-tolerant DLBA and salt-sensitive DH1 carrot varieties exposed to salt stress. For abbreviations see Table 1.

The obtained results indicate that the tolerance of DLBA variety to salt stress is partially determined constitutively, however, the exposition to saline soil triggers a physiological response that is more evident in the root than in the leaves. The most noticeable adaptations of carrot plants to stress conditions are osmotic adjustments and activation of antioxidant system (Fig. 3). Salinity induces a high increase in the content of osmoprotective proline in the salt-tolerant variety. Antioxidant protection from damages caused by oxidative stress is maintained by low-molecular glutathione. This coenzyme and antioxidant molecule appear to play an important role by ensuring efficient operation of the ascorbate–glutathione cycle and regulation of redox potential. Antioxidant protection of the intracellular space in carrot roots is also supported by enzymatic system (increased peroxidase activity with no support of catalase). Hydrogen peroxide generated under salt stress can be indicated as a signalling molecule initiating antioxidative response.

## Materials and methods

### Plant material and experimental design

Salt stress was applied to plants of two carrot (*Daucus carota* L. ssp. *sativus*) varieties, DH1 and DLBA. The DH1 variety is a doubled haploid population of the Nantes western type carrot^82^ and it is sensitive to salinity. DLBA is a local variety, exhibiting tolerance to salinity, grown on saline soil in the Fars region in Iran. The experiment was established in a plastic tunnel with plants growing in solid containers (60 × 40 × 41 cm length/width/height) without drainage and was set up in a completely randomized design with five replications for each variety grown in the saline or non-saline (control) soil as described previously by Smoleń et al.^44^. In brief, 24 plants were growing per treatment combination per replication in the control soil of EC 0.2 dS/m composed of peat substrate and sand, and in the saline soil of the same composition but with EC adjusted with NaCl to 3.0 dS/m. To ensure uniform seed germination and unaffected seedlings development, a 10 cm layer of non-saline soil (EC 0.2 dS/m) was placed on the top of the saline soil, and to which seeds were sown. Tap water was used for irrigation for 8 weeks after sowing, then 100 mM NaCl solution was applied 22 times every 2–3 days (on average, 0.6 L per container per day) to increase salinity of the top layer of the soil and to maintain a salinity level in the container. After 102 days of vegetation, the final EC of the soil was 3.15 dS m^−1^. Plants growing in the control soil were irrigated with tap water at the same time and using the same volume as stressed plants. The final EC of the control soil after vegetation was 0.22 dS m^−1^. Two weeks before harvest, ten leaves from different plants were collected for each treatment combination in each replication and used for determination of the state of leaf hydration. At harvest, leaves and storage roots of ten plants per combination were collected separately, frozen in liquid nitrogen, grinded and stored at − 80 °C until subjected to biochemical analyses.

The study complies local, Polish and international regulations. Seeds (DH1 variety) were obtained from prof. P.W. Simon (Univ. of Wisconsin-Madison and USDA-ARS, Madison, WI, USA), and from the Univ. of Agriculture in Krakow carrot collection (DLBA variety, accession id: DLBA).

### Leaf relative water content (RWC)

Fully developed leaves were used to determine the state of tissue hydration. The leaves were cut off from the plant, weighed and immediately placed in plastic bags with water so that only the petiole was submerged. The bags were sealed and kept in the dark at room temperature for 8 h. The leaves were then weighed again and dried at 60 °C to obtain the dry matter of the tissue. Calculations of RWC (%) values were made based on the following formulas:

1 $${\text{RWC }} = \left( {{\text{FW}} - {\text{DW}}} \right) /\left( {{\text{TW}} - {\text{DW}}} \right) \, \times { 1}00$$

where: FW—fresh weight; DW—dry weight; TW—tissue weight at maximum hydration.

### Proline and free amino acids contents

Proline content was assayed according to Bates et al.^83^ with modification. Tissue samples were homogenized in 3% aqueous sulphosalicylic acid and centrifuged (3000×*g*, 10 min, 4 °C). Extracts with acid-ninhydrin and glacial acetic acid (1:1:1) were incubated in a boiling water for 1 h, and then reaction was terminated in an ice bath. Reaction mixture was extracted with toluene, vigorously mixed and the absorbance at 520 nm of toluene phase was measured using Genesys 10 spectrophotometer (ThermoFisher Scientific, Waltham, USA). The content of free amino acids was determined with 0.2% ninhydrin in isopropanol using 80% methanolic extracts. Absorbance was recorded at 570 nm. Glycine was taken for standard solution curve determination.

### Lipid peroxidation

Lipid peroxidation was determined by measurement of MDA content described by Hodges et al.^84^. Plant tissue was homogenized in 80% ethanol and centrifuged (3000 × *g*, 10 min, 4 °C). The supernatant was incubated with 20% TCA containing 0.01% BHT, with or without 0.5% TBA at 95 °C for 25 min. Absorbance was recorded at 440, 532 and 600 nm. MDA (nmol g^−1^ FW) was calculated as:

2 $${1}0{3 } \times \, \left[ {\left( {{\text{A }} - {\text{ B}}} \right)/{157}} \right],$$

3 $${\text{where}}{:}\;{\text{ A }} = \, \left( {{\text{Abs}}_{{{532}}} - {\text{ Abs}}_{{{6}00}} } \right){\text{TBA}}^{ + } - \, \left( {{\text{Abs}}_{{{532}}} - {\text{ Abs}}_{{{6}00}} } \right){\text{TBA}}^{ - } ,$$

4 $${\text{B }} = 0.0{571 } \times \, \left( {{\text{Abs}}_{{{44}0}} {-}{\text{ Abs}}_{{{6}00}} } \right){\text{TBA}}^{ + }$$

### Hydrogen peroxide content

Hydrogen Peroxide Assay Kit (Colorimetric/Fluorometric) (ab102500, Abcam, Cambridge, UK) was used to measure H_2_O_2_ levels according to the manufacturer’s instructions. The assay is based on the OxiRed Probe reaction with H_2_O_2_ in the presence of horseradish peroxidase (HRP), which results with a colour product (Abs570).

### Antioxidative enzymes activity

Determination of POD activity was based on spectrophotometric measurements^85^. Plant material was homogenized with potassium phosphate buffer (0.067 M, pH 6.2) and centrifuged (3000×*g*, 15 min, 4 °C). Reaction mixture was consisted of plant extract, hydrogen peroxide (30 mM) and *p*-phenylenediamine (4.6 mM). The absorbance (Abs485) was read (U-2900 UV–Vis spectrophotometer, Hitachi, Tokyo, Japan) after 1 and 2 min from H_2_O_2_ addition to the reaction mix. Peroxidase activity was expressed in units of the amount of enzyme that causes the absorbance increase of 0.1 per minute.

Determination of CAT activity was done according to Aebi^86^. Plant material was homogenized in potassium phosphate buffer (0.05 M, pH 7) and centrifuged (10,000×*g*, 4 °C). Plant extract (0.2 mL) was added to 1.8 mL of 0.05 M potassium phosphate buffer (pH 7) and mixed with 1 mL of 30 mM H_2_O_2_ solution. Reaction mixture was vortexed and absorbance at 240 nm was read 5 min after the addition of H_2_O_2_. CAT activity was expressed as the amount of hydrogen peroxide (µmol) decomposed by 1 g of tissue within 1 min.

### Glutathione and ascorbate contents

Determination of glutathione and ascorbate contents was performed according to Queval and Noctor^87^. Cold extraction of the tissue was performed with the use of 0.2 N HCl. The homogenate was centrifuged at 16,000×*g* for 10 min at 4 °C and neutralized with 0.2 M NaOH to pH 5–6. Glutathione was measured by the recycling assay: 10 µl of neutralized extract was added to 0.1 ml of 0.2 M NaH_2_PO_4_ (pH 7.5), 10 mM EDTA, 10 µl of 10 mM NADPH, 10 µl of 12 mM 5,5-dithiobis(2-nitro-benzoic acid) (DTNB), and 60 µl of water. The reaction was initiated by the addition of 10 µl glutathione reductase and the increase of absorbance at 412 nm was monitored for 5 min. Standards of 0 to 1 nmol GSH were run in the same manner. Total ascorbate content was measured in the neutralized extracts after incubation with 25 mM dithiothreitol (DTT). 0.1 ml of 0.2 M NaH_2_PO_4_ (pH 5.6) and 75 µl of water were added and absorbance at 265 nm was recorded. Then, 5 µl of ascorbate oxidase was added, solutions were mixed and the decrease of absorbance was monitored for 5 min. The ascorbate concentration in the samples was calculated on the basis of the standard curve.

### Phenolic compounds contents

Total phenolics content was determined with Folin–Ciocalteu (F–C) assay^88^. Tissue was homogenized in ice-cold 80% methanol and centrifuged at 13,000×*g* for 5 min at room temperature. Incubation of extracts with 10% F–C reagent and 700 mM Na_2_CO_3_ was performed for 2 h. Absorbance was measured at 765 nm immediately after incubation. Standard curve was prepared with chlorogenic acid. Total phenolics content was expressed as chlorogenic acid equivalents using the regression equation.

Phenylpropanoids, flavonols and anthocyanins contents were estimated spectrophotometrically^89^. Phenolics were extracted from frozen samples with the use of 80% methanol and centrifuged at 10,000×*g* at 4 °C. 0.25 mL of supernatants were then mixed with 0.25 mL of 0.1% HCl in 95% ethanol and 4.5 mL of 2% HCl. The absorbances of the samples for phenylpropanoids, flavonols and anthocyanins concentrations were read at 320, 360, and 520 nm, respectively. Calculations of phenolics contents were made on the basis of standard curves, where caffeic acid, quercetin, and cyanidin 3-glucoside were used to estimate contents of phenylpropanoids, flavonols, and anthocyanins, respectively.

For HPLC analysis, 0.2 g of material was homogenized in 80% methanol (HPLC grade, Sigma-Aldrich) and then centrifuged at 5400×*g* and 4 °C for 15 min. The supernatants were diluted to the total volume of 15 mL in the above solvent. Analysis were performed using the LC-20 AD chromatograph (Shimadzu, Kioto, Japan) equipped with a CTO-10AS column oven and SPDM20A diode array detector (DAD), at 25 °C. Runs were conducted on Phenomenex Synergi 4 μm Fusion-RP 80A (250 × 4.6 mm) column, in the water (B) -methanol (A) (HPLC-grade, Sigma-Aldrich) gradient (10–100%), with the addition of H_3_PO_4_. The solvent velocity was set as 1 mL min^−1^ and sample volume accounted for 20 μL. The phenolic compounds were detected according to standards: *p*-HBA, chlorogenic acid, caffeic acid, *p*-coumaric acid, ferulic acid, sinapic acid, quercetin rutinoside, hesperidine, quercetin and naringenin. The standards were prepared in the concentration of 1 or 10 mg mL^−1^ in 80% methanol and kept up to 2 weeks at − 18 °C in the darkness. The individual compounds were detected in the range between 254 and 520 nm. The quantitative analysis was done at 325 nm^90^.

### Statistical analysis

Two independent extractions and reactions were performed for each sample, and the calculated mean values were subjected to statistical analyses using STATISTICA 13.0 software (StatSoft, Tulsa, OK, USA). Data were analysed by applying a two-way analysis of variance followed by the Newman–Keuls post hoc multiple comparison test. The tested effects or differences between means were considered significant at the significance level P < 0.05. Hierarchical cluster analysis was performed by applying Ward amalgamation to Euclidean distances of standardized data; then clustering using two-way joining was performed. Principal component analysis (PCA) was performed to visualize relationships between biochemical parameters and plant populations (control and exposed to salt stress) in two-dimensional plots.

## Data Availability

The datasets generated and/or analysed during the current study are available from the corresponding author on reasonable request.
